# Conventional and PEGylated Liposomes as Vehicles of *Copaifera sabulicola*

**DOI:** 10.3390/pharmaceutics15020671

**Published:** 2023-02-16

**Authors:** Ian M. R. Blanco, Raquel de Melo Barbosa, Julita M. P. Borges, Silvio A. B. Vieira de Melo, Ramon dos Santos El-Bachá, César Viseras, Patricia Severino, Elena Sanchez-Lopez, Eliana B. Souto, Elaine Cabral-Albuquerque

**Affiliations:** 1 Industrial Engineering Program, Polytechnic School, Federal University of Bahia, Salvador 40210-630, Bahia, Brazil; 2 Department of Pharmacy and Pharmaceutical Technology, School of Pharmacy, University of Granada, Campus of Cartuja s/n, 18071 Granada, Spain; 3 Department of Science and Technology, State University of Southwestern Bahia, Salvador 45083-900, Bahia, Brazil; 4 Laboratory of Neurochemistry and Cell Biology, Department of Biochemistry and Biophysics, Institute of Health Sciences, UFBA, Salvador 40170-110, Bahia, Brazil; 5 Biotechnological Postgraduate Program, Tiradentes University, Aracaju 49010-390, Sergipe, Brazil; 6 Department of Pharmacy, Pharmaceutical Technology and Physical Chemistry, Faculty of Pharmacy and Food Sciences, University of Barcelona, 08007 Barcelona, Spain; 7 Institute of Nanoscience and Nanotechnology (IN2UB), University of Barcelona, 08007 Barcelona, Spain; 8 Unit of Synthesis and Biomedical Applications of Peptides, IQAC-CSIC, 08034 Barcelona, Spain; 9 UCIBIO—Applied Molecular Biosciences Unit, MEDTECH, Laboratory of Pharmaceutical Technology, Department of Drug Sciences, Faculty of Pharmacy, University of Porto, 4050-313 Porto, Portugal; 10 Associate Laboratory i4HB, Institute for Health and Bioeconomy, Faculty of Pharmacy, University of Porto, 4050-313 Porto, Portugal

**Keywords:** copaiba oil, liposomes, antitumor activity, *Copaifera* spp., astrocytes

## Abstract

Traditional medicine uses resin oils extracted from plants of the genus *Copaifera* for several purposes. Resin oils are being studied to understand and profile their pharmacological properties. The aim of this work was to prepare and to characterize conventional and pegylated liposomes incorporating resin oils or the hexanic extract obtained from *Copaifera sabulicola* (copaiba) leaves. The cytotoxic effect of these products was also investigated. Conventional and stealth liposomes with copaiba extract showed similar average diameters (around 126 nm), encapsulation efficiencies greater than 75% and were stable for 90 days. A cytotoxicity test was performed on murine glioma cells and the developed liposomes presented antiproliferative action against these cancer cells at the average concentration of 30 μg/mL. Phytochemicals encapsulated in PEGylated liposomes induced greater reduction in the viability of tumor cells. In addition, bioassay-s measured the cytotoxicity of copaiba resin oil (*Copaifera sabulicola*) in liposomes (conventional and PEGylated), which was also checked against pheochromocytoma PC12 cells. Its safety was verified in normal rat astrocytes. The results indicate that liposomes encapsulating copaiba oil showed cytotoxic activity against the studied tumor strains in a dose-dependent fashion, demonstrating their potential applications as a chemotherapeutic bioactive formulation.

## 1. Introduction

Brazil has the richest flora in the world of plant species of biological interest that can be exploited for several biological uses, such as for anti-inflammatory and antiparasitic activities [1,2,3,4]. Studies published over the last 20 years have shown that the genus *Copaifera* produces a resin oil that is rich in sesquiterpenes and diterpenes and has recognized leishmanicidal, anti-inflammatory and antitumor activities [5]. In 1972, the Food and Drug Administration (FDA) approved copaiba oil for human use, after studies involving its safety for uses in cosmetic, food and pharmaceutical applications. Eleven studies were included, and several copaiba species were studied. Regardless of its species, all studies showed that copaiba oil presented a bactericidal and/or bacteriostatic effect in vitro [6]. Recently, Genesi et al. (2023) [7] reported promising results of chitosan films containing 0.5% copaiba oil for wound healing in adult female rats.

A biological evaluation of the action of copaiba resin oil and its fractions showed that they have antitumor activity against melanoma cells in both in vivo and in vitro models [8]. Its plant metabolites, such as diterpenes [9], have shown antitumor efficacy by inducing apoptosis in brain tumor cells [10].

One limitation of the use of such biomaterials in pharmaceutical formulations is their easy oxidation and low solubility in an aqueous medium. The bioactive molecules present in copaiba oil are nonpolar in nature and can readily oxidize when exposed to light and at temperatures higher than 30 °C [11,12,13].

To improve the chemical and physical stability of plant bioactives, as well as their solubility in aqueous media, their incorporation in controlled release particulate systems was proposed [14]. Liposomes and nanoparticles of lipid and natural polymers have shown great potential in improving the solubility and increasing the therapeutic efficacy of lipophilic drugs, including oils and hydroalcoholic extracts of plants and microalgae sources [15,16,17]. Liposomes are spherical colloidal vesicles based on a lipid bilayer structure, similar to the cell membrane [18]. As one of the preponderant drug delivery systems, liposomal formulations have several advantages for drug administration, as they can load amphiphilic bioactives, increase the solubility of poorly water-soluble molecules, protect the payload and promote a modified release profile with site-specific targeting potential [19,20,21]. Liposomes have a high structural versatility and can be applied successfully in medical–pharmaceutical areas [22]. Several studies in the literature show the added value of liposomes over polymer particles for antitumor applications [23,24,25,26]. However, systemic clearance by the reticuloendothelial system (RES) and poor selectivity for tumor sites are still the main limitations for most conventional liposomes that lead to short systemic circulation time and low cellular uptake. To overcome these problems, various modified liposomes were developed, for example, by their surface tailoring with polyethylene glycol (PEG; PEGylation) or with cationic moieties [27,28,29,30,31].

PEG is a nontoxic, water-soluble and biocompatible polymer with a high mobility and water-binding capacity [32]. PEGylation can not only improve the physical and biological properties of liposomes, but it can also protect them from clearance due to their recognition by RES, shielding and prolonging their residence time in the blood stream [13].

The antioxidant activity of extracts of the *Hibiscus sabdariffa* flowers incorporated in liposomes was greater than in their free-form [33]. The development of poly (lactic-co-glycolic acid) nanoparticles with the organic extract of *Phytolacca decandra* roots allowed an increase in antineoplastic activity [34]. The developed solid lipid nanoparticles containing allantoin and copaiba oil improved the antifungal activity compared to nonencapsulated copaiba oil, showing a similar result compared to commercial antifungal agents [35]. More recently, copaiba oil was proposed in combination with aloe vera in the production of chitosan films for wound healing [7,36]. The in vivo experiments run in female adult rats confirmed that tested 0.5% copaiba-loaded chitosan films were superior to commercial dressing formulations in their capacity to regenerate epithelium and vessel neoformation.

The literature shows the potential of liposomes in antitumor formulations. The *Zanthoxyllum tingoassuiba* essential oil was encapsulated in liposomes, obtaining appraisable activity against murine glioma compared to free essential oil [37]. It was also observed that Doxil^®^ (doxorubicin in liposomal vector) was also active against refractory ovarian cancer, which was later approved by the FDA for its treatment [38]. Recently, it was approved for the treatment of breast cancer in the USA and for the treatment of multiple myeloma in combination with bortezomib (Velcade, Takeda Pharmaceuticals MA, USA) in Europe and Canada [38,39].

In this context, the objective of this work was the development and characterization of conventional and PEG-containing liposomes as carriers of the *Copaifera sabulicola* fraction (extract or resin oil). The biological properties of liposomes were explored by evaluating the cytotoxicity of these formulations against pheochromocytoma tumor PC12 cells and by the study of their safety in normal astrocytes.

## 2. Materials and Methods

### 2.1. Materials

Natural soy phosphatidylcholine (PC) was acquired from LIPOID, INC-PHOS-125 PHOLIPON^®^ 90G (Ludwigshafen, Germany) and PEG laurate (LP) from LIPO chemicals INC (Warren, NJ, USA). Trypan blue and NaHCO_3_ were obtained from Sigma-Aldrich (St. Louis, USA). DMEM and a Serum Bovine Fetal antibiotic (100 U/mL penicillin 100 U/mL streptomycin) were acquired from Gibco (Paisley, Scotland). For the cell-viability assay, 3-(4,5-dimethylthiazol-2-yl)-2,5-diphenyltetrazolium bromide (MTT), sodium dodecyl sulfate (SDS), dimethyl formamide (DMF), dimethyl sulfoxide (DMSO) and bovine serum albumin were from Sigma-Aldrich (St. Louis, MO, USA). Other reagents and organic solvents were of analytical grade. Sterile water was used throughout the formulations.

### 2.2. Methods

#### 2.2.1. Extract Preparation

The hexanic extract (EXT) obtained from the leaves of *C. sabulicola* and its resin oil (CP) were kindly donated by Dr. Eudes Velozo, a professor at the Federal University of Bahia. Raw material, EXT and CP were obtained and characterized following the procedures described previously [40,41].

#### 2.2.2. Preparation of Liposomes

Conventional and PEGylated liposomes have the presence of phosphatidylcholine and copaiba resin oil in common. Only PEGylated liposomes have PEG-laurate. Empty conventional and PEGylated liposomes were also prepared for comparison purposes, and were used as standards. Multilamellar liposomes (MLV) were prepared according to the classical lipid film hydration methodology, as described by Bangham [42]. Briefly, soybean phosphatidylcholine, PEG and copaiba essential resin oil were dissolved in chloroform (10 mL). The active substance (CP or EXT) lipid mass ratio was approximately 1:10. The solvent was evaporated under reduced pressure at room temperature (~25 °C). The lipid film was hydrated with freshly prepared sodium phosphate buffer (PBS—0.01M) and pH 7.4 under constant stirring. The vesicles acquired the unilamellar form (SUV) from MLV dispersions by tip ultrasonication at 40 kHz for 30 min (60 s on and 60 s off) in a Vibra-Cell ultrasonic processor (Sonics and Materials; Newtown, CT, USA). Finally, the liposomes were stored in a refrigerator (4–8 °C) for stability studies.

#### 2.2.3. Characterization of Liposomes

##### Size Distribution and Superficial/Surface Charge

Analysis of the size and zeta potential of the samples in water was performed by the Zeta nanosizer ZS equipment (Malvern INC, Worcestershire, United Kingdom). The samples were measured 24 h after preparation. The physical stability of the dispersions was monitored by the size distribution measures over time (7, 14, 30 and 90 days after production). Liposomes’ size (diameter) prior to the sonication process was also measured in Mastersizer 3000 (Malvern INC., Worcestershire, United Kingdom) by laser diffraction.

##### Liposomal Dispersion Morphology

The samples were prepared in copper grids covered with film forming. The dispersed vesicle (~0.5 mL) was deposited on the grids and allowed to stand for 10 min. They were then stained with 1% phosphotungstic acid for 1 min. One day later, the samples were visualized and photographed under a transmission electron microscope (TEM) Zeiss EM 902A, Orius SC 1000 (1 × 300), (Gatan, Munich, Germany) at an acceleration voltage of 50 kV.

##### Encapsulation Efficiency of CP and EXT

Liposomes were separated from free bioactive molecules using the Amicon^®^ Ultra Centrifugal Filters device (10 kDa of pore filtration unit) (Merck KGaA, Darmstadt, Germany). The amount of the encapsulated CP or copaiba EXT was determined by gas chromatography (GC). The calibration curves were performed in triplicate, using pure CP and pure EXT as standards. A linear equation was obtained from the main peak areas (two major components). The bioactive encapsulated CP or EXT was extracted and injected into the chromatograph after the disruption of the liposomes. Liposomal samples were disrupted with dichloromethane in a volume ratio of 1:3 (sample: solvent). They were then vortexed and centrifuged for 20 min at 20,000× *g*. The organic phase containing the extract or encapsulated resin oil was collected and analyzed by GC (Gas Chromatograph Mass Spectrometer- Shimadzu- Model: QP-2010 (Shimadzu Scientific Instruments, Kyoto, Japan). Column: EN5MS (30 m × 0.25 mm × 0.25 µm) SGE Analytical Science (Trajan, Victoria, Australia). The encapsulation efficiency (*EE*) was obtained by the percentage ratio between the mass of the encapsulated resin oil or extract (*Mop*) and the initial mass of CP or EXT (*MI*) (Equation (1)).

(1) $$EE\left(\%\right)=\frac{\left[Mop\right]}{\left[MI\right]}\times 100$$

#### 2.2.4. Cell Cultures

##### Cell Line Cultures

The rat glioma C6 or rat pheochromocytoma PC12 cell lines (Banco de Células do Rio de Janeiro (BCRJ), Rio de Janeiro, Brazil) grew in culture medium (DMEM) supplemented with NaHCO_3_ (44 mM), 10% (*v*/*v*) bovine fetal serum (SFB) and antibiotics (100 IU/mL penicillin: 100 µg/mL streptomycin) (Gibco, Paisley, Scotland) in a humidified atmosphere with 5% CO_2_ at 37 °C. The medium was renewed every two days, until cell confluence.

Twenty-four hours before beginning the experiment, the cells (C6 or PC12) were detached from the bottles by trypsin action, and the cell count was performed by using trypan blue (Sigma Aldrich, St. Luis, USA). For testing, cells were seeded in 96-well plates at a density of 3.1 × 10^4^ cells/cm^2^.

##### Primary Culture of Rat Astrocytes

The primary culture of astrocytes was obtained from newborn Wistar rats [43,44] in agreement with the Ethics Committee on Animal Use, Institute of Health Sciences—Federal University of Bahia (UFBA; CEUA/ICS 067/2014). The newborn animals (0–48 h) underwent decapitation followed by asepsis of the skulls. After craniotomy, the cerebral cortices were removed and dissected in a sterile field. The meninges were discarded and the cortices were subjected to mechanical cell dissociation with a glass Pasteur pipette in cold DMEM supplemented with antibiotics (100 μg/mL streptomycin −100 IU/mL penicillin). After centrifugation at 2000× *g* for 10 min at 4°C, cells were suspended in a medium, distributed in 75 cm^2^ bottles and kept at 37 °C in a humid environment with 5% CO_2_. After 48 h the culture was shaken vigorously in order to detach microglial cells before exchange of the medium. Cells were washed with 0.01 M PBS and pH 7.4 (two times) and the medium was replaced. The medium was exchanged every 48 h until cell confluence was attained. For tests, cells were detached using trypsin and seeded in 96-well plates at a density of 3.1 × 10^4^ cells/cm^2^.

##### Cell Treatment

Twenty-four hours after plating cells in 96-well plates, the medium was changed and CP or copaiba leaf extract, either nonencapsulated or encapsulated into conventional or PEGylated liposomes, were tested in C6 and PC12 cells, as well as in astrocytes. Samples were added directly to the culture medium at final concentration ranges between 0.2 and 30 µg/mL. Cells were exposed for 72 h at 37 °C in a humid atmosphere with 5% CO_2_. The control group for treatments with liposomes constituted of 5% (*v*/*v*) empty liposomes in the medium. The control group composed of 0.05% dimethyl sulfoxide was used as a solvent. After 72 h of treatment, the cell viability was evaluated by using 3-(4,5-dimethylthiazol-2-yl)-2,5-diphenyltetrazolium (MTT) (Sigma Aldrich, St. Louis, USA).

##### Cytotoxicity Assay

The cytotoxicity of the tested formulations was measured by the metabolizing ability of mitochondrial dehydrogenases to convert MTT into formazan [45]. After treatments, cells were incubated with 100 µL of medium with MTT (1 g/L) for 2 h. After a period of incubation of 2 h, cells were lysed and formazan crystals were dissolved with 100 µL of 20% (*w*/*v*) sodium dodecyl sulfate in a solution of 50% (*v*/*v*) *N,N*-dimethyl formamide in reagent grade water at pH 4.7 and left overnight at 37 °C [46]. The absorbance was measured at 595 nm using a microplate reader (THERMO PLATE, Thermo Fisher Scientific, Waltham, Massachusetts, United States). The results were expressed relative to the control group, which was considered as having 100% viability. The results are shown as cell viability, which corresponds to the mitochondrial dehydrogenase activity. All cultures were also examined by phase contrast microscopy with a green filter (LabomedR TCM400 Microscope, Tokyo, Japan) to evaluate morphological alterations and cellularity.

#### 2.2.5. Statistical Analysis

Data were expressed as central tendency and dispersion measurements in accordance with the frequency distribution, analyzed by the D’Agostino-Pearson normality test, skewness (normal considered between −1 and 1) and the Kurtosis (normal in the range between −1.5 and 1.5) calculation. Parametric or nonparametric tests were chosen according to the distribution. The parametric unpaired Student *t*-test or one-way ANOVA (followed by Dunnett’s test for multiple comparison) were used in the case of normal distributions and the nonparametric Mann–Whitney test or the Kruskal–Wallis test (followed by Dunn’s test) for multiple comparisons in the case of non-normal distributions. Significant differences were considered for *p* < 0.05. From data obtained in the concentration–response curves, it was possible to determine the concentration that was able to kill 50% of cells (EC_50_). The software used was GraphPad Prism, version 5.00 for Windows (GraphPad Software, San Diego, CA, USA). The nonlinear regression calculations were performed using equation models from the software library, considering a good fit for R^2^ higher than 0.9.

## 3. Results

### 3.1. Physicochemical Characterization of Liposomes

**Figure 1:**
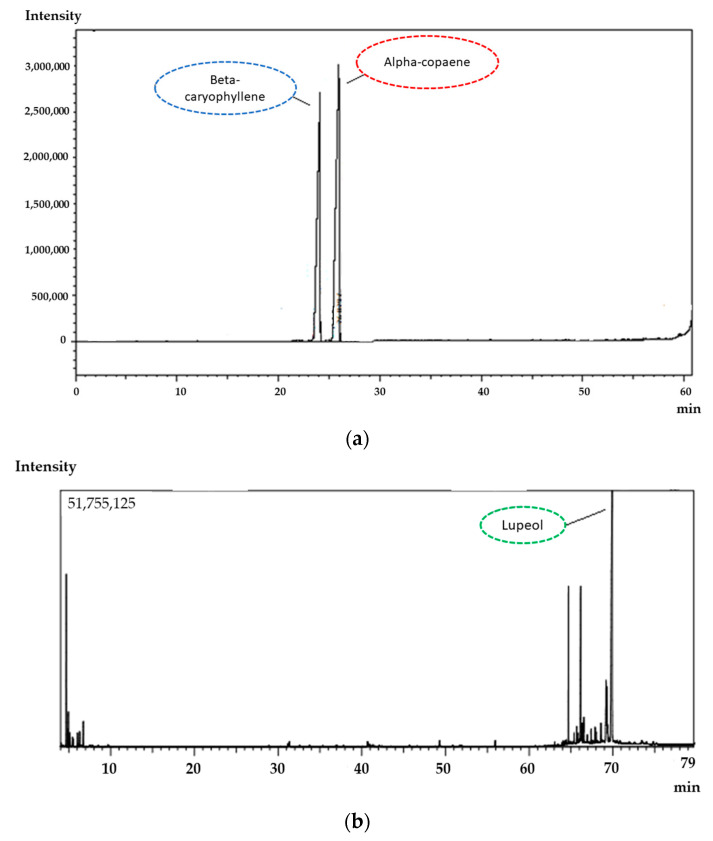
Chromatographic profile of the CP (**a**) and extract (**b**) by CG highlighted the major components, alpha-copaene, beta-caryophyllene and lupeol.

The chromatographic profile of CP samples shows two major components: alpha-copaene and trans-caryophyllene (corresponding to ~42% (area) of the total resin oil composition) (Figure 1a). In addition, the EXT of *C. sabulicola* leaves showed as major component lupeol, corresponding to 40% (area) of the total composition. These results agree with the literature since studies of the chromatographic profile of the copaiba CP also showed trans caryophyllene as the major component [47,48,49]. The composition of the EXT of *C. sabulicola* leaves showed the presence of significant sesquiterpenes, as observed in other *Copaifera* species [50]. The spectroscopic assays performed by GC-MS showed 12 bioactive substances in the copaiba oleoresin representing 76.1% of the volatile fraction, while for leaf extract, 14 bioactive substances were recovered representing 53.3% of the volatile fraction of our previously published research work [40].

The results of the size distribution, zeta potential and encapsulation efficiency of CP and copaiba leaves EXT in liposomes are shown in Table 1. The produced liposomes depicted nanometric sizes. The produced liposomes had similar mean diameters (approximately 126 nm). Conventional liposomes had a relatively larger particle diameter than the PEGylated ones, showing statistical difference between the particle sizes in the different formulations (*p* > 0.05). The zeta potential of all the developed liposomes was close to neutrality as expected. Table 2 shows the mean diameter of the liposomes monitored over the course of 90 days. The mean diameter of the liposomes had a very small deviation, with no statistical significance (*p* < 0.05).

CP and EXT were successfully incorporated into conventional and PEGylated liposomes. In all cases, the encapsulation efficiency was greater than 80%. These data suggest that there was only a small loss of CP and EXT during liposome production, indicating the suitability of the proposed method to obtain loaded liposomes with CP and EXT. The loss of payload was attributed to the evaporation during liposomal production or prior the chromatography analysis which involves extracting the bioactive by disrupting liposomes with chloroform. In general, the encapsulation efficiency of bioactive substances in the liposomes essentially depends on the nature and concentration of the phospholipid, and the procedure adopted to obtain liposomes. The location of the bioactive within the liposomes’ structure depends on the partition coefficient between the aqueous and lipid phases, whereas the maximum amount of bioactive to be incorporated depends on the complete solubility in both phases and the type of liposomal structure (multilamellar versus unilamellar) [51]. These liposomal systems can enhance the interaction between the oil and the target cells suspended in aqueous media and the protection against the oxidation of terpenes, improving the chemical stability of the latter.

The incorporation of CP and copaiba EXT in the dispersions did not alter the diameter of the conventional liposomes nor of their PEGylated counterparts, due to the low oil content and the extract used. Similar results were obtained by incorporating coconut oil into liposomes in the mass ratio of approximately 1:5 coconut oil/egg phosphatidylcholine, resulting in 80% encapsulation efficiency [52]. Yang et al. (2009) [52] observed that liposomes containing coconut oil (123 nm) were comparable to empty liposomes (113 nm). This result is similar to that reported by Gibis et al. [33], who encapsulated polyphenolic grape seed extract by high pressure homogenization and obtained liposomes of a mean diameter of less than 200 nm showing stability for more than 120 days.

**Figure 2:**
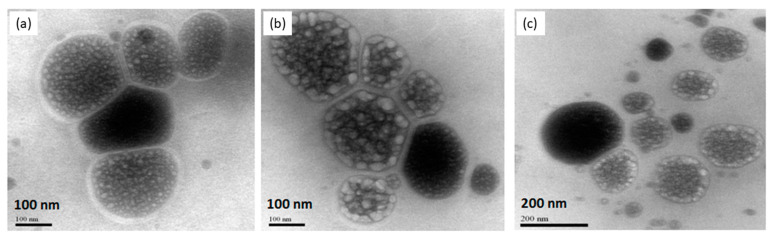
Transmission Electron Microscopy of CP entrapped into liposomes. PC + PL (**a**), PC + LP + CP (**b**), PC + EXT (**c**). Scale bars: 100 nm (**a**,**b**) and 200 nm (**c**).

Liposomes were also characterized for morphology by transmission electron microscopy. The photomicrograph of the different liposomal dispersions showed the typical spherical form of the liposomes (Figure 2). Microscopic analysis confirmed the nanometric size of the obtained liposomes.

### 3.2. Biological Properties of Liposomes

#### 3.2.1. Cell Viability in Murine Glioma (C6)

**Figure 3:**
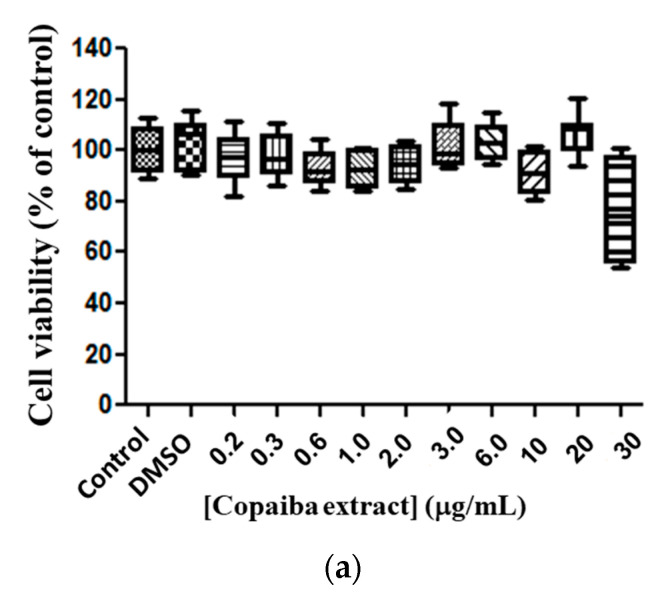

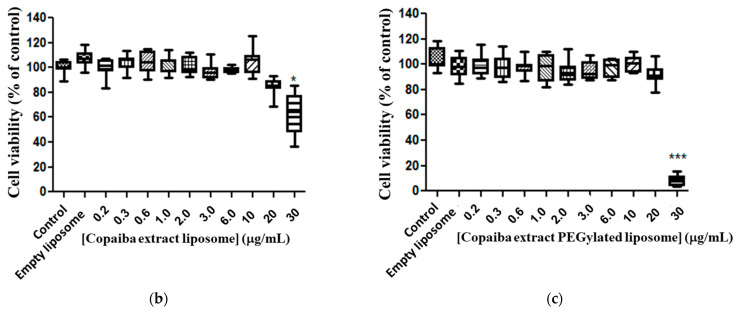
Cytotoxicity of copaiba extract in glioma line cell (C6). Exposure for 72 h, at doses of 0.2–30 µg/mL. Copaiba extract (**a**), copaiba extract in liposome conventional (PC + CP) (**b**), copaiba extract in PEGylated liposome (Phospholipon, lipopeg and copaiba extract—PC + LP+ EXT) (**c**). Statistical significance is shown as (*), *p* < 0.001 (***) *p* < 0.0001 compared with the control, (One-way ANOVA followed by Dunnett’s multiple comparison test). Test conducted in triplicate.

The free copaiba CP and EXT, and both liposomes (conventional and PEGylation) with CP or with EXT, were tested in line murine glioma (C6) after 72 h of exposure to evaluate the cell viability. The copaiba EXT at the tested doses did not alter the viability of the tumor line (C6) when compared to the control (Figure 3a), meaning that no cytotoxicity was reported. When analyzing the copaiba EXT encapsulated in conventional liposomes, toxicity at a dose of 30 μg/mL (*p* < 0.01) was observed, presenting a 30% reduction in cell viability compared to the control (Figure 3b). The EXT-loaded PEGylated liposomes presented a median EC_50_ value of 24.89 μg/mL (variation of 25.06–27.12 μg/mL) and a 93% reduction in cell viability of glioma compared to the control (Figure 3c), suggesting that the formulation of copaiba extract in PEGylated liposomes has a more significant effect on tumor cells.

**Figure 4:**
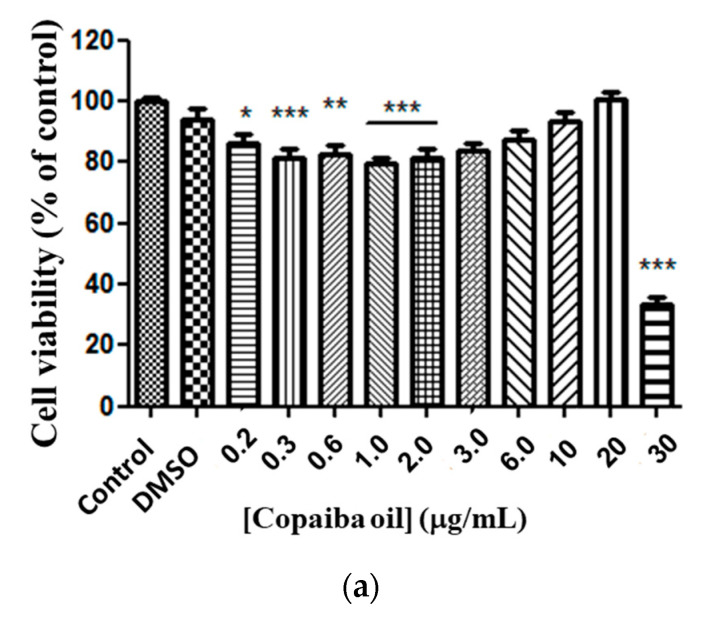

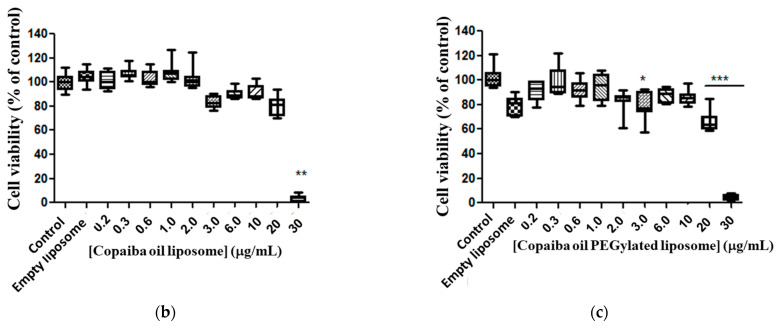
Copaiba oil cytotoxicity in glioma line cell (C6). Free CP (**a**); copaiba oil in conventional liposome (PC + CP) (**b**); CP in PEGylated liposomes (Phospholipon and lipopeg) (**c**) after exposure for 72 h, at doses 0.2–30 µg/mL. Statistical significance shown as (*) *p* < 0.01, (**) *p* < 0.001 and (***) *p* < 0.0001 compared with the control (one-way ANOVA followed by Dunnett’s multiple comparison test). n = 3.

Free CP showed moderate toxicity against the glioma cell line at doses from 0.2 to 2.0 μg/mL, compared to the control (Figure 4a). There was no significant difference between doses of 3.0 to 20 μg/mL compared to the control. However, a 67% reduction (*p* < 0.0001) in cell viability at a dose of 30 μg/mL was observed compared to the control (Figure 4a). By analyzing the response of cell murine glioma exposure to CP in conventional liposomes, no statistical difference in the responses between doses was observed, except for 30 μg/mL, and for median EC_50_ 23.13 μg/mL ratio (22.53–24.93 μg/mL) (Figure 4b). A cytotoxic response was observed for copaiba oil-loaded PEGylated liposomes at doses of 3.0, 20 and 30 μg/mL and a statistical significance compared with the control (Figure 4c) and median EC_50_ 21.65 μg/mL (21.23–21.76 μg/mL) was determined.

At the dose of 30 μg/mL there was a 98% reduction in cell viability compared to the control, killing the tumor cells in almost entirely. However, CP in PEGylated liposomes showed a slightly higher efficacy in terms of antitumor activity in the tested lineage (reduction of 98% in cell viability) when compared to the extract in the same formulation, doses and tested lineage (93%).

#### 3.2.2. Cytotoxicity of Liposomes with CP (Conventional and PEGylated) in PC12

**Figure 5:**
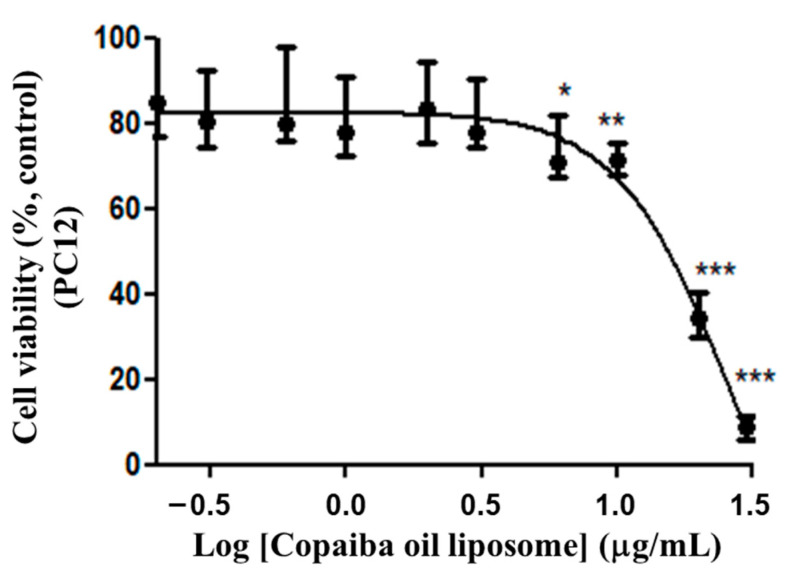
Dose–response curve of PC12 cells to CP-loaded conventional liposomes. Treatment at the dose of 0.2 to 30 μg/mL of copaiba oil in conventional liposome for 72 h. The graph represents the experiment closest to the median of the EC_50_ 15.57 μg/mL. Data are represented by a non-normal distribution in terms of the median and variation, analyzed by Kruskal–Wallis followed by Dunn’s comparison test. Statistical significance: (*) *p* < 0.01, (**) *p* < 0.001 and (***) *p* < 0.0001 compared to the control with 0.05% empty liposomes.

The response of PC12 cells to different doses of CP in conventional and PEGylated liposomes was evaluated. The results indicated that the liposomes loading CP showed cytotoxic activity in the studied strain in a dose-dependent fashion. The EC_50_ value, corresponding to the median of the remaining values, was 13.78 μg/mL (variation: 9.44–19.84 μg/mL, n = 6). The graph representing the results, being a value close to EC_50_ of 15.57 μg/mL (Figure 5), was obtained from the series of two triplicates. The dose–response curve was constructed from nonlinear regressions with the experimental data and the calculation of EC_50_ value according to Equation (2):

(2) $$V=\left\{\frac{130.04}{\left[1+10\left(2.09LogC-2.97\right)\right]}\right\}-47.58$$

$$R^{2}=0.9415$$

where *V* corresponds to the relative cellular viability compared to the control and *C* corresponds to the concentration of the resin oil.

**Figure 6:**
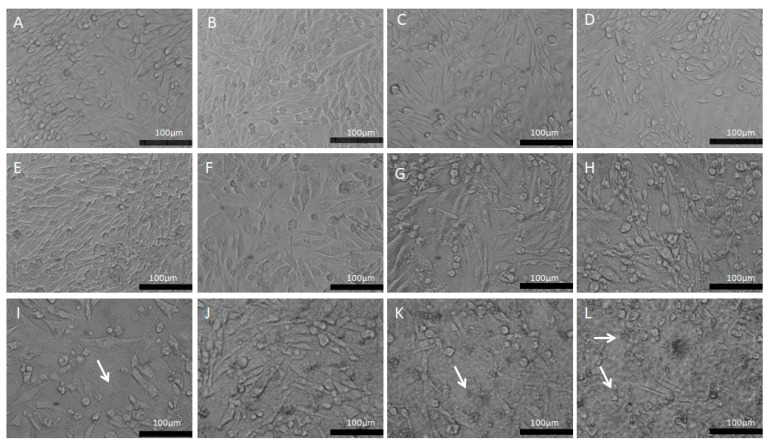
Morphological aspects of the PC12 line exposed to CP in conventional liposomes (0.2–30 μg/mL). (**A**)—Control: PC12 in DMEM medium; (**B**)—PC12 in DMEM medium treated with 0.5% empty liposomes. (**C**)—PC12 in DMEM with 0.2 μg/mL; (**D**)—PC12 in DMEM with 0.3 μg/mL; (**E**)—PC12 in DMEM with 0.6 μg/mL; (**F**)—PC12 in DMEM with 1 μg/mL; (**G**)—PC12 in DMEM with 2 μg/mL; (**H**)—PC12 in DMEM with 3 μg/mL; (**I**)—PC12 in DMEM with 6 μg/mL; (**J**)—PC12 in DMEM with 10 μg/mL; (**K**)—PC12 in DMEM with 20 μg/mL; (**L**)—PC12 in DMEM with 30 μg/mL. The white arrows represent cell fragments. Phase contrast microscopy, bar equivalent to 100 μm.

The morphological changes were observed through a phase contrast microscope (Figure 6) showing cells in control conditions (A and B) with fusiform or rounded phenotypes, preserving the cellular monolayer and maintaining homogeneous characteristics. The morphological changes were highlighted from the doses above 6 μg/mL (Figure 6E), where there was a clear presence of pycnotic cells and cell fragments, as well as greater spacing between the cells, showing the cytotoxic effect of the copaiba resin oil. This morphological appearance overlaps with the minimum cytotoxic concentration (MCC) which was 6 μg/mL (range of: 6–20 μg/mL), with a difference of 46.13% (*p* < 0.05) compared to the control group.

**Figure 7:**
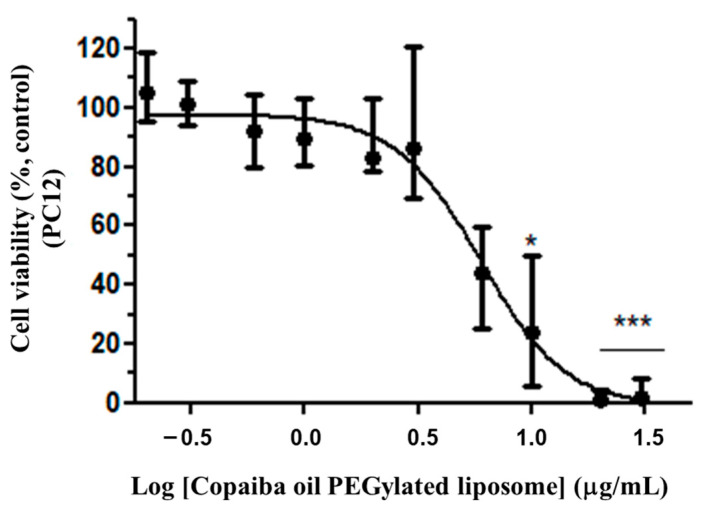
Cytotoxicity of CP-loaded PEGylated liposomes in PC12. Treatment at the dose of 0.2 to 30 μg/mL of PC in PEGylated liposomes for 72 h. The graph represents the experiment closest to the median EC_50_ value of 5.79 μg/mL. Data are represented by a non-normal distribution in terms of the median and variation, analyzed by Kruskal–Wallis followed by Dunn’s comparison test. Statistical significance: (*) *p* < 0.01 and (***) *p* < 0.0001 compared to the control with 0.05% empty liposomes.

Cell viability evaluation was performed on the PC12 cells treated with CP-loaded PEGylated liposomes. The cytotoxicity was more evident in the representative EC_50_ value of 5.79 μg/mL (Variation: 4.66–6.54 μg/mL; n = 3) (Figure 7) conducted in triplicate. The nonlinear regression analysis was originated from the dose response curve and the calculation of the EC_50_ value was obtained from the relative cell viability in comparison to the control and concentration of the resin oil, represented by *C* as in Equation (3):

(3) $$V=\left\{\frac{99.042}{\left[1+10\left(1.798LogC-13.82\right)\right]}\right\}$$

$$R^{2}=0.9306$$

**Figure 8:**
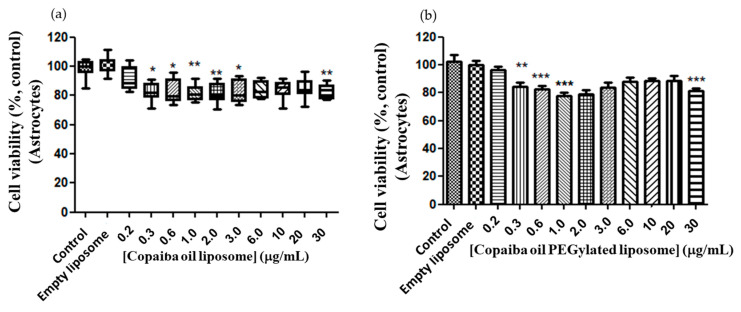
Dose–response curve of astrocytes to conventional and CP-loaded PEGylated liposomes. Treatment at the dose of 0.2 to 30 μg/mL of copaiba oil in conventional (**a**) and PEGylated (**b**) liposomes for 72 h. (**a**)—Data are represented by a non-normal distribution in terms of the median and variation, analyzed by Kruskal–Wallis followed by Dunn’s comparison test. (**b**)—Data are represented by a normal distribution in terms of the mean, analyzed by Turkey and all pars columns compared. Statistical significance: (*) *p* < 0.01, (**) *p* < 0.001 and (***) *p* < 0.0001 compared to the control with 0.05% empty liposomes.

In view of the antitumor response profile of conventional and CP-loaded PEGylated liposomes, it was necessary to evaluate the response of the astrocytes under the same experimental conditions in which the cells of the PC12 and C6 lines cells were submitted. Astrocytes were tested in the interassay test with PC12, starting from the same dilution of CP in conventional and PEGylated liposomes under the same experimental conditions. Similar dose–response curves were observed in astrocytes in both conventional and PEGylated liposomes (Figure 8a,b), with a minimum inhibitory dose of 0.3 μg/mL (with a relative minimum survival of 72% (*p* < 0.05).

**Figure 9:**
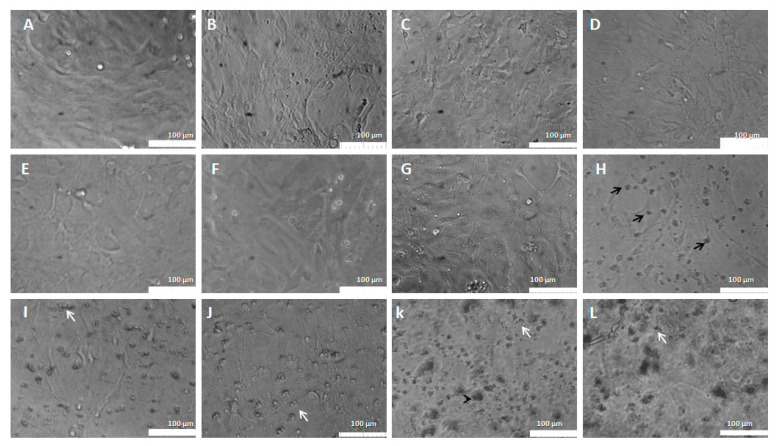
Morphological aspects of astrocytes treated with CP in conventional liposomes (0.2–30 μg/mL). (**A**)—Control: Astrocytes in DMEM medium. (**B**)—Astrocytes in DMEM medium with 0.5% empty liposomes. (**C**)—Astrocytes in DMEM medium with 0.2 μg/mL. (**D**)—Astrocytes in DMEM medium with 0.3 μg/mL. (**E**)—Astrocytes in DMEM medium with 0.6 μg/mL. (**F**)—Astrocytes in DMEM medium treated with 1 μg/mL. (**G**)—Astrocytes in DMEM medium with 2 μg/mL. (**H**)—Astrocytes in DMEM medium with 3 μg/mL. (**I**)—Astrocytes in DMEM medium with 6 μg/mL. (**J**)—Astrocytes in DMEM medium with 10 μg/mL. (**K**)—Astrocytes in DMEM medium with 20 μg/mL. (**L**)—Astrocytes in DMEM medium with 30 μg/mL. The black arrows represent cells in the carotid cortex and the white arrows represent cell fragments.

Control cells showed a normal morphology, polygonal aspect and confluent layer (Figure 9A). Moderate astrocytic reactivity was observed in cells treated with 5% empty PEGylated liposomes (Figure 9B). Astrocytes treated with conventional liposomes bearing CP exhibited morphological changes from the dose of 2 μg/mL CP in conventional liposomes. Astrogliosis is observed in cells treated with the dose of 2 μg/mL of CP in conventional liposomes with a fusiform and elongated morphology (Figure 9G). In the images of cells treated with doses 3, 6 and 10 μg/mL, carotid can be seen in some cells on the monolayer in addition to apparent cellular suffering (Figure 9H,I,J) at a dose of 20 and 30 μg/mL of cellular fragments (white arrows) (Figure 9K,L).

**Figure 10:**
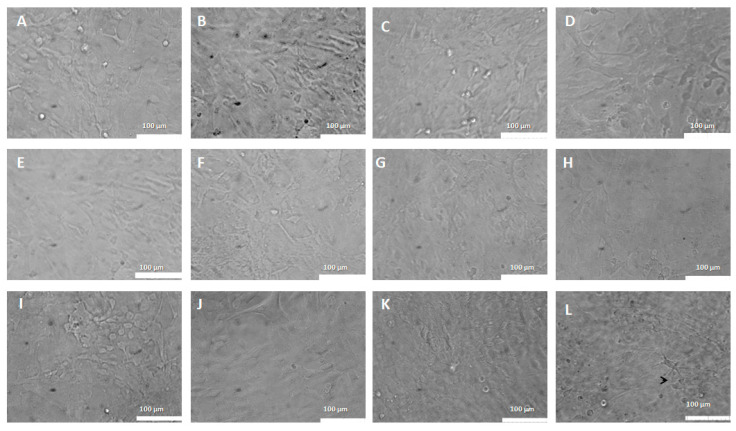
Morphological aspects of astrocytes treated with CP-loaded PEGylated liposomes (0.2–30 μg/mL). Astrocytes treated with copaiba oil in PEGylated liposomes at various doses. (**A**)—Control: Astrocytes in DMEM medium. (**B**)—with 0.5% of empty liposomes. (**C**)—0.2 μg/mL; (**D**)—0.3 μg/mL. (**E**)—0.6 μg/mL. (**F**)—1 μg/mL. (**G**)—2 μg/mL. (**H**) =3 μg/mL. (**I**)—6 μg/mL. (**J**) =10 μg/mL. (**K**)—20 μg/mL. (**L**)—30 μg/mL. Confocal microscopy, 100 μm bar.

When analyzing the morphology of astrocytes treated with CP-loaded PEGylated liposomes, cells with a normal, polygonal appearance were observed as well as confluence in the monolayer of the control (Figure 10A) and moderate astrocytic reactivity in cells treated with 5% of empty PEGylated liposomes (Figure 10B). No morphological changes were seen in cells treated with CP-loaded PEGylated liposomes at doses of 0.2 to 2 μg/mL (Figure 9C–K). Discrete astrocyte reactivity and cell agglomeration were observed on the monolayer treated with 30 μg/mL of CP-loaded PEGylated liposomes (Figure 10L).

Liposomes have been used in several pharmacotherapies, including cancer therapy [47]. *C. sabulicola* CP at the highest dose tested (30 μg/mL) had an increased antitumor effect in glioma (67% reduction in cell viability) than the leaf extract of the plant, which showed no difference when compared to control cells. The antiproliferative activity has already been evidenced by the components of other plants of the same species, such as *Copaifera langudorfii* against human mammary adenocarcinoma [53] and CP of *Copaifera multijuga* against melanoma cells, in vivo and in vitro models [8]. In behavioral tests, the dose (200 mg/kg, ip) of CP increased the anxiety of animals and reduced locomotion and exploratory activity by unknown mechanisms, indicating the presence of phytochemicals in the central nervous system [54]. A study with phytochemicals of *C. reticulata* CP showed that the most present metabolites are β-caryophyllene (37%) and β-bisabolene (14.5%) and that they may be responsible for anti-inflammatory and neuroprotective activities in models of excitotoxic injury in the motor cortex of adult rats. Authors injected a dose (400 mg/kg) of copaiba CP after the damage, evidenced by a significant reduction of neurotrophic infiltration and microglial activation [55].

In a comparative study between the species *Copaifera cearensis* Huber ex Ducke, *Copaifera reticulata* Ducke and *Copaifera multijuga* Hayne, β-caryophyllene was identified as the bioactive responsible for the anti-inflammatory action of these plant species [5]. These species come from different Brazilian regions, varying in phytochemical composition and biological response [5]. However, in human glioblastoma lines, MO59J, U343 and U251, the phytochemical of *Copaifera langsdorffii*, namely (-)—copalic acid, showed antiproliferative activity in these cells, with EC_50_ of 68.31, 222.50 and 275.20 μg/mL, respectively [53]. There are few studies involving *Copaifera sabulicola* and no studies involving the CNS.

By testing the conventional and PEGylated liposomal formulations and the properties of the copaiba bioactive (CP and EXT) as a possible antitumor agent, it was observed that in the PEGylated liposomal formulation there is a greater reduction in the viability of the tumor cells, thus providing evidence that the phytochemicals are more bioavailable when compared to the drug encapsulated in conventional liposomes. Liposomes’ delivery of drugs has shown efficacy by facilitating solubility and reducing side effects and toxicity on normal cells [55]. This refers to a nanotechnology used to deliver chemotherapeutics, increasing the bioavailability of the bioactive and its selectivity in the target tissue [47].

Many works were performed using liposomes in cancer therapy. Several liposomal formulations using different anticancer agents demonstrated better efficacy and minimal toxicity compared to the free drug [56,57]. However, the use of liposomal bioactives is still rarely found in the literature. The present work describes the potential use of copaiba bioactives encapsulated in conventional and PEGylated liposomes as anticancer agents, analyzing their potential against pheochromocytoma PC12 cells and verifying the safety in their use, using normal glial cells, namely astrocytes.

Regarding the possible antitumor action of copaiba resin oil (*C. sabulicola*), the result obtained here from the concentration that kills 50% of the PC12 cells (EC_50_) of 13.78 μg/mL suggests that the CP encapsulated in liposomes are cytotoxic against this mouse pheochromocytoma tumor line. Since PC12 cells are neuroendocrine and bear similarity to neuronal cells, it is possible that the resin oil has an effect on tumors of the nervous system.

This result agrees with findings described in the literature about the antineoplastic effect of essential resin oil (freeform), which evaluated the oral administration of *C. multijuga* essential resin oil and its fractions against Erlich tumor in mice (murine mammary adenocarcinoma) at doses of 100, 150 and 200 mg/kg. This found that after 10 days of inoculation of the tumor in the paw and peritoneum, treatment with the resin oil significantly reduced ascites volume and inhibited the increase of inflammatory mediators such as PGE2, nitric oxide and TNF in the fluid [58]. Then, indicative of powerful resin oil action against the invasive tumor, the increase of the paw volume where the tumor was inoculated was apparently inhibited and there was no significant difference between the doses.

Terpenes are considered bioactive compounds that can help in the development of new and efficient antitumor agents, given their cytotoxic properties related to apoptosis induction. Most chemotherapeutic agents exhibit only proapoptotic effects. In a glioblastoma model, Irrera et al. 2020 [59] demonstrated that the antiproliferative effects of β-caryophyllene could be blocked by a CB2 receptor antagonist. Beta-caryophyllene, one of the compounds present in the CP, is responsible for the antiproliferative effect and proapoptotic effects (exhibited via DNA “ladder” and caspase-3 activation) in tumor cell lines, while there was no apoptosis induction in normal cell lines. The apoptotic process is a cascade reaction, carried out and regulated by specialized cellular machinery, namely a family of cysteine proteases called caspases. The appearance of these caspases seems to be the biochemical event that defines a cellular response as apoptosis more than any other. The caspase family can be divided into initiator caspases (caspase-8 and caspase-9) and the executioner caspases (caspase-3, -6 and -7). An apoptotic death stimulus activates the initiator caspases which, in turn, activate the executioner caspases. The active executioners promote apoptosis by cleaving cellular substrates, which induces the morphological and biochemical features of apoptosis [60].

Moreover, PEGylated liposomes actively target strategies for central nervous system delivery of anticancer drugs across the blood–brain barrier (BBB) is basically divided into adsorptive-mediated transcytosis (AMT) and receptor-mediated transcytosis (RMT). Pegylated liposomes encapsulating CP or EXT are RMT-based systems. This strategy relies on liposomal ligand interaction with the very specific receptor-mediated transport system in the BBB. C6 cells appear to have high efficiency of internalization of conventional and PEGylated liposomes with CP, therefore pegylated liposomes with CP show similar activity in comparison to conventional ones and more than the free CP. Different behavior was observed for conventional liposomes containing the extract. Probably, conventional liposomes with EXT showed lower internalization efficiency in comparison to PEGylated one [61]. The action mechanisms for the enhanced efficacy could be explained by the increased EXT uptake and the apoptosis induced through initiating the apoptotic enzymes and the relevant apoptotic gene-expressed proteins (caspases).

A recent work published by Dahiya et al. (2022) demonstrated that liposomes possess unique physicochemical properties for utilization in drug delivery for targeting brain tumors [62]. Liposome functionalization with peptides, proteins and sugars are effective strategies for receptor targeting, enabling their permeation through the BBB, and specifically delivering a therapeutic agent to the diseased site.

Despite the enormous potential and extensive research efforts, the mechanisms of action of bioactives from copaiba (free and entrapped into liposomes) concerning healthy astrocytes and PC12 pheochromocytoma cells are still limited due to several challenges associated with their developmental complexities. They require more studies (*in silico,* in vitro and in vivo in-depth studies) to propose the action of bioactives present in copaiba (loaded or not in liposomes) in the selectivity of astrocyte cells line. Interestingly, a formulation of the resin oil of imiquimod and copaiba is promising for the treatment of basal cell skin cancer. Despite this evaluation only concluding the best form of delivery, it contributed to corroborating the important antiproliferative properties of resin oil adjuvant which is already a well-established drug [63].

## 4. Conclusions

In conclusion, copaiba CP and EXT from *C. sabulicola* leaf were efficiently incorpo-rated (75% encapsulation efficiency) into both conventional and pegylated nanometric liposomes. The cellular viability responses in murine glioma showed that the resin oil was more efficient than the leaf extract. The copaiba CP in PEGylated liposomes showed antiproliferative action on glioma cells 1.06-fold more than when incorporated into conventional liposomes and 1.5 times more in the free-form for 30 µg/mL. In addition, CP showed selectivity for PC12 and showed non-toxicity in astrocytes.

## Figures and Tables

**Table 1:** Median diameter, zeta potential and encapsulation efficiency of liposomes.

Samples	Size (nm)	ZP (mV)	EE %
PC	131.3 (127.3–135.3)	+0.40 (0.31–0.54)	n.a.
PC + CP	106.4 *** (95.1–117.7)	+2.3 **(0.22–0.38)	90.10 (86.80–91.30)
PC + EXT	131.4 (125.3–140.4)	+0.59 ** (0.52–0.67)	85.60 (83.70–89.40)
PC + LP	145.8 ** (134.2–157.6)	+0.83 (0.76–0.90)	n.a.
PC + LP + CP	101.6 *** (98.7–104.8)	+2.35 ** (2.29–2.41)	85.60 (83.70–89.40)
PC + LP + EXT	110.5 *** (107.4–116.1)	+0.86 (0.81–0.94)	84.40 (81.89–86.30)

Note: Data presented a non-normal distribution; data are shown as the medians and ranges andwere analyzed by the Mann–Whitney test. Statistical significance: (**) *p* < 0.001 and (***) *p* < 0.0001 compared to the empty liposomes. (n = 6). EE % (n = 3). n.a.—not applicable (empty samples).

**Table 2:** Median diameter (nm) of the liposomal dispersion monitored over the course of 90 days.

	Time (Days)			
Liposomes	1st	15th	30th	90th
PC	131.3 (127.5–135.6)	124.6 (119.3–130.0)	130.2 (121.5–139.9)	130.9 (127.6–134.3)
PC + CP	145.8 (124.2–157.6)	137.2 (121.2–153.3)	150.7 (146.6–154.8)	143.2 (142.3–144.2)
PC + EXT	131.4 (125.3–138.6)	122.7 (120.2–124.6)	128.5 (126.4–131.6)	138.4 (130.8–142.9)
PC + LP	106.4 (95.18–117.7)	118.5 (108.8–128.3)	119.7 (113.9–125.5)	118.5 (117.8–119.2)
PC + LP + CP	101.6 (98.71–104.8)	117.0 (115.7–118.4)	128.0 (124.5–131.4)	128.3 (127.8–128.7)
PC + LP + EXT	110.5 (108.6–116.1)	102.7 (99.76–104.8)	101.0 (98.68–106.6)	104.0 (102.9–107.8)

## Data Availability

Available from authors.
